# The Hot Air Syringe

**Published:** 1871-06

**Authors:** 


### THE HOT AIR SYRINGE.

In the spring of 1855, the late Prof. Elisha Townsend, Dr. Samuel S. White, and the writer were together at the residence of Dr. J. M. Brown. Dr. T., with his usual enthusiasm, expressed a wish for some one to tell him how to thoroughly dry a cavity, preparatory to filling. Said he: "I don't mean, how to dip most of the water out, but I mean *dry—absolutely dry.*" His earnestness acted like inspiration on at least one of his auditors. The writer of this replied, "Why, Doctor, throw a current of warm air into it." "That will do it," said he, "get up your instrument." The same week, the writer and Dr. Taft demonstrated the principle with an instrument imperfectly—indeed but partially constructed. The experiment being satisfactory, the writer, having more leisure than Dr. T., constructed a complete instrument, which was regularly used and exhibited on suitable occasions. At the meeting of the American Dental Convention, at Hope Chapel, New York, in 1856, this instrument was borrowed by a dentist who forgot to return it.

From this instrument, with suggested modifications, the drawing was made, which appeared first in Toland's *Dental Catalogue*, and afterward in Taft's *Operative Dentistry*. Previous to the Hope Chapel meeting the instrument had been described in the DENTAL REGISTER, over the signature of Taft & Watt.

The instrument did not receive the attention it deserved. It should be in the hands of every operative dentist. The rubber dam enables us to keep cavities dry, and this instrument, in connection with it, makes tooth-filling a pastime, as compared with former modes of operating.

The instrument has been so much neglected that Dr. Moffett has invented it over again; and, if it would hasten its general introduction, it ought to be invented every six months. It is now on sale, at a moderate price, in the depots generally. W.

